# Physiological and Biochemical Performances of Menthol-Induced Aposymbiotic Corals

**DOI:** 10.1371/journal.pone.0046406

**Published:** 2012-09-27

**Authors:** Jih-Terng Wang, Yi-Yun Chen, Kwee Siong Tew, Pei-Jei Meng, Chaolun A. Chen

**Affiliations:** 1 Graduate Institute of Biotechnology, Tajen University, Pingtung, Taiwan; 2 National Museum of Marine Biology and Aquarium, Pingtung, Taiwan; 3 Institute of Marine Biodiversity and Evolution, National Dong Hwa University, Pingtung, Taiwan; 4 Biodiversity Research Center and Taiwan International Graduate Program (TIGP)-Biodiversity, Academia Sinica, Taipei, Taiwan; 5 Institute of Oceanography, National Taiwan University, Taipei, Taiwan; King Abdullah University of Science and Technology, Saudi Arabia

## Abstract

The unique mutualism between corals and their photosynthetic zooxanthellae (*Symbiodinium* spp.) is the driving force behind functional assemblages of coral reefs. However, the respective roles of hosts and *Symbiodinium* in this endosymbiotic association, particularly in response to environmental challenges (e.g., high sea surface temperatures), remain unsettled. One of the key obstacles is to produce and maintain aposymbiotic coral hosts for experimental purposes. In this study, a simple and gentle protocol to generate aposymbiotic coral hosts (*Isopora palifera* and *Stylophora pistillata*) was developed using repeated incubation in menthol/artificial seawater (ASW) medium under light and in ASW in darkness, which depleted more than 99% of *Symbiodinium* from the host within 4∼8 days. As indicated by the respiration rate, energy metabolism (by malate dehydrogenase activity), and nitrogen metabolism (by glutamate dehydrogenase activity and profiles of free amino acids), the physiological and biochemical performances of the menthol-induced aposymbiotic corals were comparable to their symbiotic counterparts without nutrient supplementation (e.g., for *Stylophora*) or with a nutrient supplement containing glycerol, vitamins, and a host mimic of free amino acid mixture (e.g., for *Isopora*). Differences in biochemical responses to menthol-induced bleaching between *Stylophora* and *Isopora* were attributed to the former digesting *Symbiodinium* rather than expelling the algae live as found in the latter species. Our studies showed that menthol could successfully bleach corals and provided aposymbiotic corals for further exploration of coral-alga symbioses.

## Introduction

The unique mutualism between corals and their photosynthetic zooxanthellae (*Symbiodinium* spp.) underpins ecological success of corals in shallow and oligotrophic seawater. However, this association is highly vulnerable to rising seawater temperatures. A rise of only 1∼2°C above the summer average under moderate to high irradiance will likely be enough to disrupt the symbiotic relationships by causing the symbionts to be expelled from the host, precipitating so-called ‘coral bleaching’ [1], [2]. Coral bleaching events are known to further cause a breakdown [1]–[4] or phase shift [5]–[7] in coral reefs. These situations are predicted to worsen with time if the increase in seawater surface temperatures cannot be slowed [8], [9].

In order to understand if corals can survive the coming stressful environments, the mechanisms underlying coral bleaching have been intensively studied (reviewed in Weis [10]). It is widely accepted that reactive oxygen species (ROS) generated by *Symbiodinium* photoinhibition and/or mitochondrial dysfunction in the host can cause breakdown of the symbiotic association [10]–[12]. However, the comparative susceptibility of coral hosts and *Symbiodinium* to thermal stresses is not completely understood. In studies of symbionts, cultured and freshly isolated *Symbiodinium* (FIS) was widely used to explore the symbiont physiology. Different physiological performances, such as the photosynthesis capability under thermal stress, of FIS or cultured *Symbiodinium* were also revealed at the clade or subclade levels [13]–[16]. In contrast, studies on physiological responses of aposymbiotic coral hosts are limi'ted due to a lack of suitable protocols.

Several methods were used to deplete *Symbiodinium* from cnidarian hosts, including cold shock (e.g., 4°C) [17]–[19], a high seawater temperature (e.g., 33°C) [20], and 3-(3,4-dichlorophenyl)-1,1-dimethylurea (DCMU) treatment [21], but few of them generated healthy aposymbiotic coral hosts which could be used for further studies. Aposymbiotic corals induced by high seawater temperatures either take a long time and need antibiotics treatment [20] or result in high coral mortality [22]. High-temperature treatment might also implant a heat experience in corals which might influence the performance of bleached corals in thermal-tolerance studies. On the other hand, bleaching corals with DCMU requires high light intensities (e.g., 70% of ambient insolation) and large volumes of seawater (ca. 1000 L) to maintain the animals, which prevents laboratories without ample seawater supplies and outdoor facilities from conducting coral-bleaching experiments. Consequently, physiological and biochemical studies on aposymbiotic hosts in *Symbiodinium*-cnidarian symbioses are mostly confined to sea anemones [19], [23]–[26] and aposymbiotic larvae from limited coral species [27], [28]. Nevertheless there are still gaps in applying the knowledge obtained from sea anemones to corals when the coral skeleton, calcification, and surface and endoskeletal microbes should be taken into account [29]. Therefore, a general method needs to be developed to prepare as many species of aposymbiotic corals from adult individuals to conduct comparative analyses among coral species.

Menthol is a cyclic terpene alcohol which is usually used to anesthetize cnidarians in marine biological studies [30]. This compound was occasionally found to bleach *Symbiodinium*-associated corals and sea anemones during anesthetization (unpublished data). Despite menthol's toxicity to corals being unclear, menthol was found to be less toxic to an aquatic invertebrate (*Daphnia magna*) for which the 24-h 50% lethal concentration (LC_50_) is 37.7∼71.0 mg L^−1^ [31]. In this study, therefore, extant corals from two major lineages, respectively of robust and complex clades, were used to explore a workable procedure to prepare aposymbiotic corals from adult colonies. Furthermore, the physiological and biochemical performances of the aposymbiotic coral hosts were examined, and their comparability to their symbiotic counterparts was evaluated. Feeding an artificial diet was also used to examine the effect of exogenous nutrients on maintaining physiological and biochemical performances of aposymbiotic coral compared to their symbiotic counterparts.

## Materials and Methods

### Experimental organisms

*Isopora palifera* (robust clade) and *Stylophora pistillata* (complex clade) were respectively collected from 3 and 7 m in depth within Kenting National Park, Taiwan (21°55′54″N, 120°44′45″E) between October 2010 and November 2011. Coral colonies were transferred to the laboratory within 3 h in an aerated plastic box, and maintained in an aquarium (90×45×45 cm) equipped with illumination [12: 12-h light-dark regime and ca. 50 µmol photons m^−2^s^−1^ photosynthetically active radiation (PAR)], temperature control (25°C), filtration (EHEIM, Germany), and a protein skimmer. Corals were acclimatized to laboratory conditions for 1 week before conducting the experiments.

### Bleaching coral in menthol-artificial seawater (ASW) and feeding trial

Menthol-induced coral bleaching was examined by incubating an *Isopora* fragment (ca. 5×5 cm in size) in a crystallizing dish (125×65 mm, Corning, Kaiserslautern, Germany) containing 300 ml menthol supplemented with ASW (Instant Ocean, Aquarium Systems, Sarrebourg Cedex, France) with aeration and under standard illumination as described above at 25°C. The menthol/ASW medium was prepared by diluting a 20% (w/v) menthol stock (in ethanol) with ASW and was used to bleach *Isopora* at concentrations of 0.19, 0.38, and 0.58 mM. Released *Symbiodinium* was collected by centrifuging the medium at 860×*g* for 3 min. The bleaching test was stopped when the coral tissue began to shrink, and the remaining *Symbiodinium* alga in *Isopora* was collected by air-blasting and centrifugation as described in a previous paper [32]. Numbers of *Symbiodinium* cells collected were counted with a Neubauer improved hemocytometer (Marienfeld Superior, Lauda-Königshofen, Germany) to determine the coral bleaching rate. Two nutrient cocktails (A and B) were used to feed bleached *Isopora* to test if nutrient supplementation was necessary to maintain the physiological and biochemical performance comparable to their symbiotic counterparts. The common supplements for nutrient A and B were 200 µg ml^−1^ cobalamin, 4 µg ml^−1^ biotin, and 10% glycerol. The amino acid supplement (see details in Table 1) to nutrient A was 10.5 mM of a free amino acid (FAA) mixture, an FAA pool mimic of that in the *Isopora* tissue, and that to nutrient B was a 10.5 mM so-called ‘essential’ FAA mixture. The 10% glycerol supplement was used to provide the coral host with organic carbon and also to increase the supplement viscosity, such that the nutrient cocktail would remain on the coral surface for awhile.

**Table 1:** Mole percentages of amino acids in the nutrient supplements for maintaining aposymbiotic *Isopora palifera*.

Amino acid	Free amino acid supplement (mol %)	
	Nutrient A	Nutrient B
Aspartate	4.2	-
Glutamate	13.5	-
Asparagine	2.0	-
Serine	2.9	-
Histidine	6.0	10.0
Glutamine	4.5	-
Glycine	1.9	-
Threonine	10.9	10.0
Arginine	5.7	-
Taurine	1.4	-
Alanine	4.2	-
Tyrosine	10.3	10.0
γ-Aminobutyric acid	2.2	-
Tryptophane	0.9	10.0
Methionine	1.5	10.0
Valine	5.0	10.0
Phenylalanine	2.7	10.0
Isoleucine	3.4	10.0
Leucine	3.9	10.0
Lysine	12.8	10.0
Total	100.0	100.0

### Determination of physiological and biochemical indices

The respiration rate was used to represent the general physiological performance of symbiotic and aposymbiotic coral hosts, which was determined in a custom-made respiration chamber (400 ml) which was connected with a BOD probe (YSI 5905, Yellow Springs, OH, USA) and a dissolved oxygen (DO) meter (YSI 52). Oxygen consumption by the coral host in the respiration chamber was continuously determined by connecting the meters to a personal computer for 15 min in darkness. The respiration rate of the coral host per se in symbiosis was determined by subtracting the dark respiration rate of an equivalent amount of *Symbiodinium* in the whole symbiotic consortium from the total oxygen consumption by the symbiotic coral. The dark respiration rate of *Symbiodinium* was determined with freshly isolated algae in a Hansatech Oxygraph System (Hansatech Instrument, Norfolk, UK).

Biochemical indices of the coral host were determined with the apparent activities of malate dehydrogenase (MDH) and glutamate dehydrogenase (GDH), and the FAA profile. MDH, one of the key enzymes in energy anabolism [33], was used to represent the energy-synthesizing capacity of the coral host. GDH and the composition of FAAs are two key factors which are usually used to reveal the nitrogen status of *Symbiodinium*-associated corals and sea anemone hosts [25], [34]. To prepare the host homogenate, a *Stylophora* branch of about 10 cm or an area of 25 cm^2^ of *Isopora* was stripped of tissues with 4°C seawater buffer [25] carried by air blasting. The resulting tissue slurry stored on ice was homogenized in a syringe and then centrifuged at 21,500×*g* for 10 min (4°C) to remove cell debris and *Symbiodinium*. The enzyme extract was immediately stored at −80°C and analyzed within 3 days. MDH activity was determined by adding 100 µl of host homogenate to 1 ml of the reaction mixture containing 80 mM imidazole-HCl buffer (pH 7.0), 100 mM KCl, 0.3 mM oxaloacetate, and 0.15 mM NADH at room temperature. The activity of GDH was determined in the aminating direction, in which 100 µl of host homogenate was incubated with 1 ml of reaction mixture containing 50 mM HEPES buffer at pH 7.4, 0.2 mM NADPH, and 10 mM α-ketoglutarate at room temperature. The enzyme activity was measured by the decrease in absorbance at 340 nm, and was expressed as nmol NAD(P)^+^ formed per milligram tissue protein per minute. Co-incubation of the host extract and reaction mixture without substrate (oxaloacetate for MDH and α-ketoglutarate for GDH) was used as a blank to confirm the specificity of the enzyme reaction, which reduced the activity by over 95%. Prior to determining FAAs in the host homogenate, samples were precipitated with 70% ethanol. FAAs in the resulting ethanol extract were quantified by a reverse-phase high-performance liquid chromatographic (HPLC) system using a pre-column derivatization method modified from Jones et al. [35]. For the HPLC analysis, the amino acids were derivatized with *o*-phthaldialdehyde and separated with a solvent delivery system (Hitachi L-2130, Tokyo, Japan), using a C18-ultrasphere column and fluorescence detector (Hitachi L-2485). The reference amino acid mixture was AA-S-18 (Sigma) supplemented with asparagine, glutamine, γ-aminobutyric acid (GABA), trypophan, and taurine. It was noted that cysteine and proline could not be detected by this technique. The protein content in the host homogenate was quantified by a protein assay kit of Bio-Rad Chemical (Hercules, CA, USA), following the manufacturer's instruction for the microassay with 0∼12 µg bovine serum albumin as the standard.

### Statistical analysis

Data in this study are presented as the mean±S.E. from numbers of different colonies. Comparisons of the host respiration rates, MDH and GDH activities, and FAA contents in the host homogenate between symbiotic and aposymbiotic coral were made using a one-way analysis of variance (ANOVA) followed by Fisher's least significance difference (LSD) test for multiple comparisons at a significance level of 0.05. For the similarity analysis of the FAA composition between symbiotic and aposymbiotic coral hosts, the mole % data of FAAs were arc cosine-transformed to meet the normality and homogeneity of variance assumptions. The similarity of the FAA compositions was compared using multidimensional scaling (MDS) ordination [36], [37]. An analysis of similarity (ANOSIM) was used to determine whether FAA profiles from different treatments separated by MDS ordination significantly differed [38]. The analyses were carried out using the computer package, PRIMER 6 [38].

## Results

**Figure 1:**
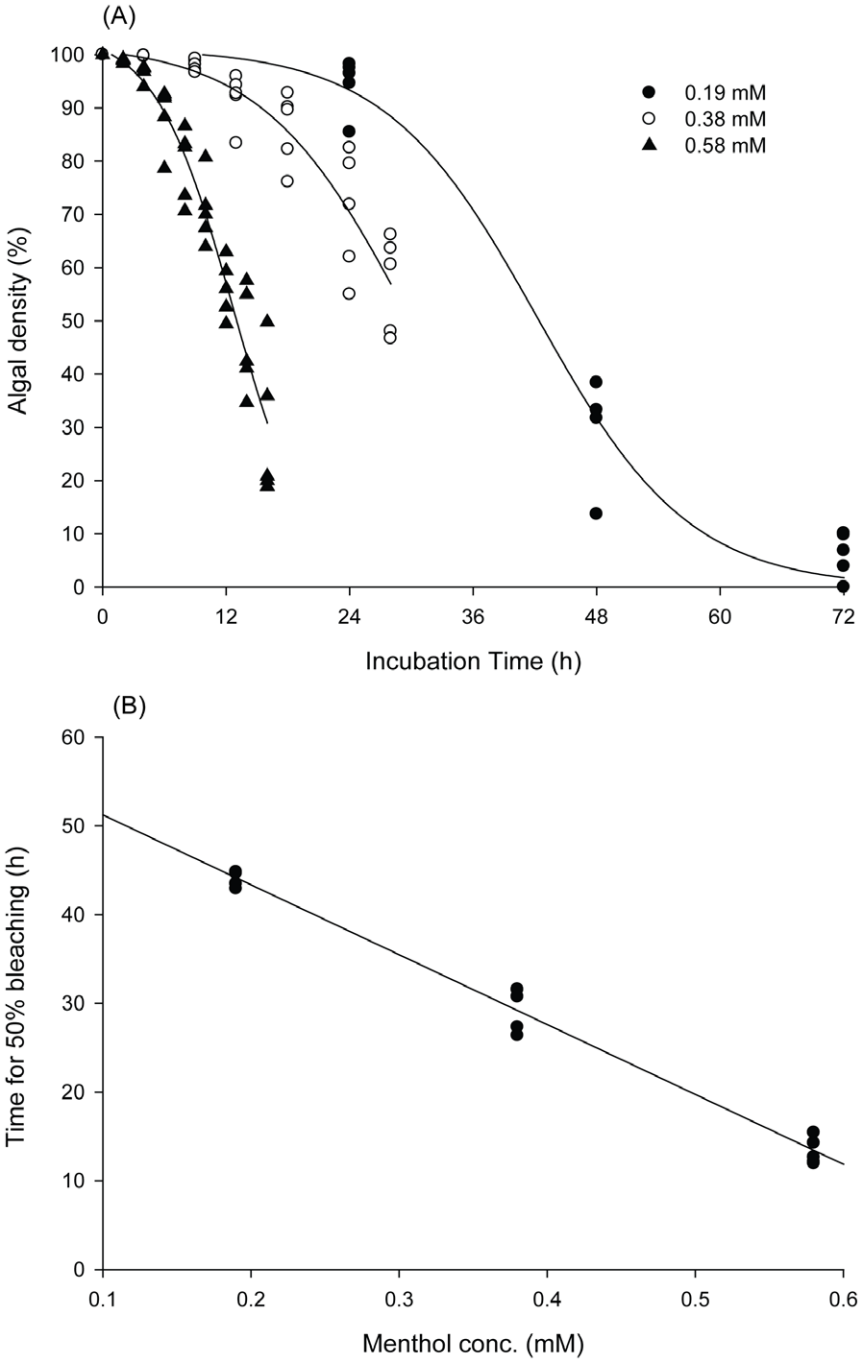
Bleaching *Isopora palifera* with menthol. (A) Decrease in the *Symbiodinium* density in treated coral with different menthol concentrations; (B) correlation between the menthol concentration and the time for 50% coral bleaching.

**Figure 2:**
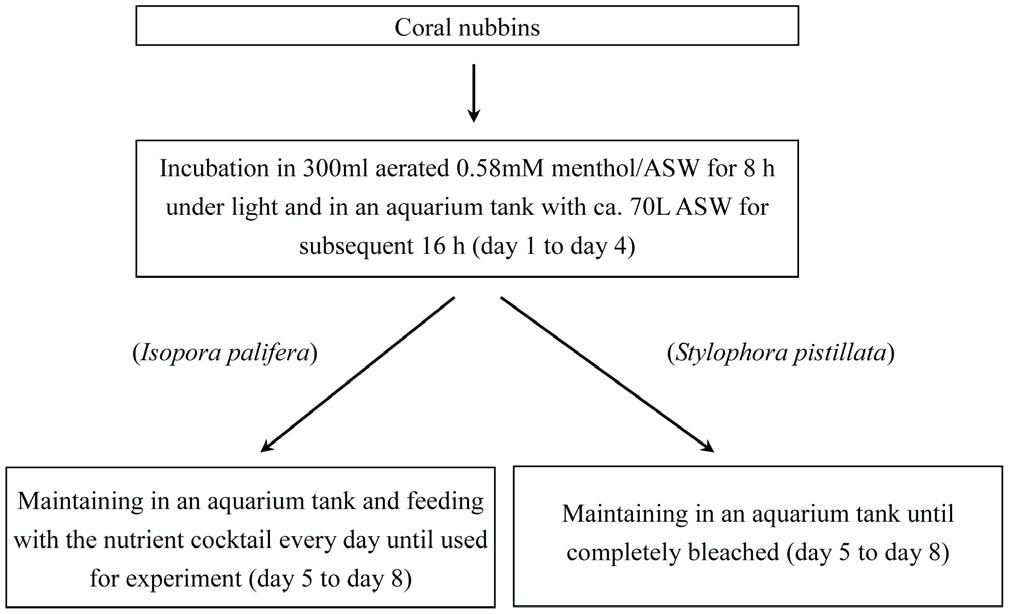
Flow diagram of the preparation of aposymbiotic *Isopora palifera* and *Stylophora pistillata*.

**Figure 3:**
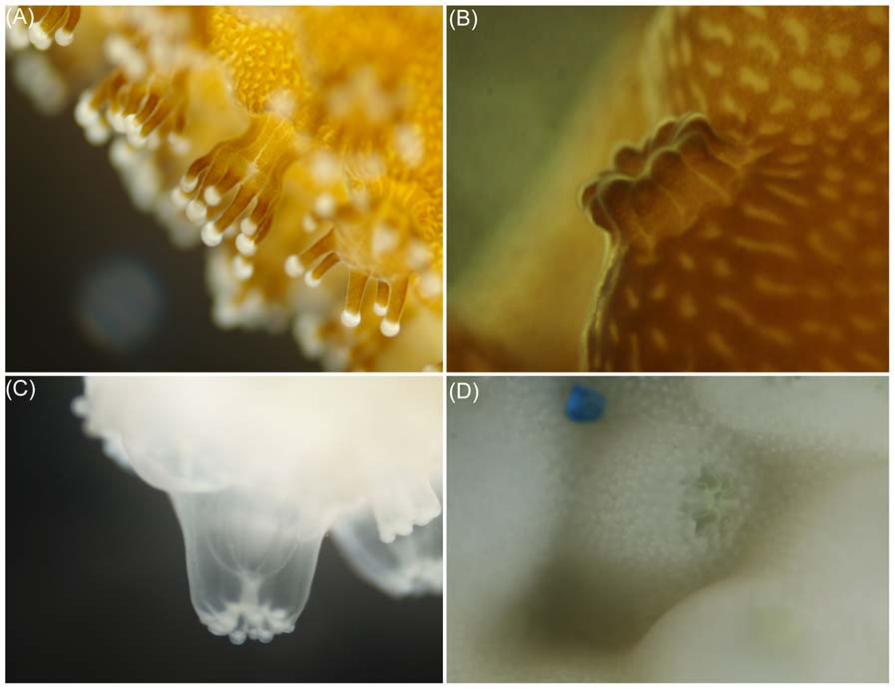
Polyps of symbiotic and aposymbiotic corals under microscopic examination. (A) and (C) are *Stylophora pistillata*; (B) and (D) are *Isopora palifera*.

When *I. palifera* was incubated in menthol-supplemented ASW, the *Symbiodinium* density in the coral declined with an increase in the incubation time, and rates of algal depletion were dependent on the concentration of menthol used (Fig. 1A). In order to examine the dose response of menthol, the decline in *Symbiodinium* density with time was converted to an equation using a curve-fitting model provided by SigmaPlot. The equation that best fit as determined by *r* ^2^ values was:
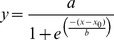
. Parameters of the equations for 0.19, 0.38, and 0.58 mM menthol treatment were: a = 101.22, b = −7.37, and x_0_ = 42.24 (*r* ^2^ = 0.979); a = 102.08, b = −7.08, and x_0_ = 29.65 (*r* ^2^ = 0.875); and a = 104.43, b = −3.79, and x_0_ = 12.71 (*r* ^2^ = 0.934), respectively. Using this equation, the times for 50% coral bleaching at different menthol concentrations were estimated and regressed on the menthol concentration used. As shown in Fig. 1B, the time for 50% coral bleaching was significantly correlated with the menthol concentration used (*p*<0.0001), and the correlation was fit to the linear regression equation: y = 59.11–78.76x (*r* ^2^ = 0.983). Although 0.58 mM menthol could bleach *Isopora* comparatively rapidly, continuous incubation at that concentration for 24 h always caused high (>80%) mortality. In order to obtain a rapid and gentle bleaching procedure, the duration of menthol treatment was reduced to 8 h following by 16 h of resting in an aquarium without menthol, and the mortality rate was significantly reduced in this way. With the protocol described in Fig. 2, 4 repeats of the above treatment/resting cycle could expel almost all *Symbiodinium* from *Isopora* and *Stylophora* (see as Fig. 3) within 4∼8 days after being maintained in an aquarium without menthol, which resulted in respective 0% and <10% mortalities in aposymbiotic *Stylophora* and *Isopora* preparations. It was also found that *Isopora* and *Stylophora* released *Symbiodinium* in different modes during menthol treatment. *Symbiodinium* released by menthol-treated *Isopora* was in a cloudy suspension and retained some PSII activity (*F_v_/F_m_* = 0.3∼0.5), but that from menthol-treated *Stylophora* aggregated into black granules which displayed no detectable PSII activity. When coral was bleached, a nutrient cocktail was fed from day 5 for aposymbiotic *Isopora*, but aposymbiotic *Stylophora* was not fed due to its physiological and biochemical performances being comparable to its symbiotic counterpart (see below). As shown in Fig. 3, the aposymbiotic and symbiotic *Isopora* and *Stylophora* displayed comparably healthy shapes to each other.

**Figure 4:**
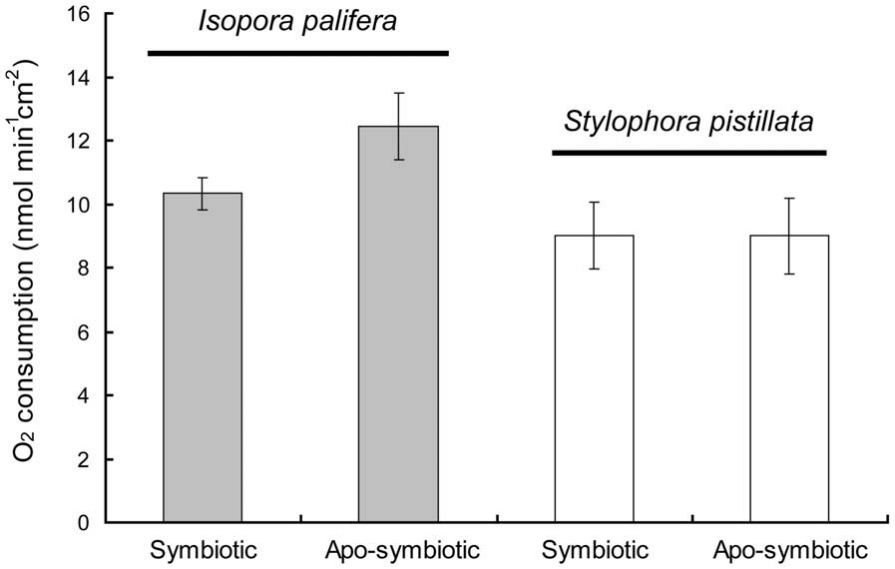
Respiration rates of symbiotic and menthol-bleached *Isopora palifera* and *Stylophora pistillata*. Aposymbiotic *I*. *palifera* with feeding was treated by supplementing animals with a nutrient cocktail containing glycerol, vitamins, and a host mimic free amino acid mixture as described in the [text/Table 1??]. Data represent the mean±S.E.

The extents of physiological and biochemical comparability between symbiotic and aposymbiotic corals were further examined. In this study, the term, aposymbiotic host, represents freshly bleached corals which were examined at 6∼10 days after menthol treatment. When comparing respiration rates, as shown in Fig. 4, those of the aposymbiotic hosts were 12.5±1.1 nmol min^−1^cm^−2^ (*n* = 5) for *Isopora* and 9.0±1.2 nmol min^−1^cm^−2^ (*n* = 5) for *Stylophora*. These data did not significantly differ from their symbiotic counterparts [10.3±0.5 nmol min^−1^cm^−2^ (*n* = 7) for *Isopora*, F_1,11_ = 3.996, *p*>0.05; and 9.0±1.1 nmol min^−1^cm^−2^ (*n* = 9) for *Stylophora*, F_1,12_ = 0.000, *p*>0.05]. Feeding aposymbiotic *Isopora* and *Stylophora* with the nutrient cocktail did not produce significant differences between the symbiotic and aposymbiotic corals (data not shown).

Biochemical indices (MDH, GDH, and the FAA pool) in the host homogenate were further examined. As shown in Table 2, GDH activity, total FAAs, and “essential” FAAs in *Isopora* were significantly reduced by 50.0% , 44.7%, and 43.7%, respectively, after bleaching (*p*<0.05). However, depletion of *Symbiodinium* produced no difference in MDH activities between the symbiotic and aposymbiotic *Isopora* (*p*>0.05). “Essential” FAAs noted here followed the definition applied to the sea anemone *Aiptasia pulchella* [19]. Levels of GDH and FAAs (total and essential) in aposymbiotic *Isopora* could be reverted to comparable levels of the symbiotic counterpart by feeding with nutrient A. However, feeding with nutrient B (containing a mixture of essential FAAs) was less effective than nutrient A in reverting GDH and FAA levels back to those of the symbiotic counterpart. Total FAA content in nutrient B supplemented-aposymbiotic *Isopora* reverted to about 73% of that in the symbiotic counterpart, but the value was still significantly lower (*p*<0.01) than that from the symbiotic counterpart. On the contrary, nutrient B supplementation reverted levels of GDH and essential FAAs in aposymbiotic *Isopora* to levels comparable to the symbiotic counterpart (*p*>0.05). Inconsistent with *Isopora*, levels of GDH activity, and total and essential FAAs displayed no significant (*p*>0.05) differences between symbiotic and aposymbiotic *Stylophora* (Table 3). However, as shown in Table 3, depletion of *Symbiodinium* from *Stylophora* still caused a significant increase in host MDH activity by 49.5% (*p*<0.005). Samples from aposymbiotic *Stylophora* with feeding were not further examined because data in Table 3 suggested that feeding might not be necessary.

**Table 2:** Contents of free amino acids and activities of malate dehydrogenase (MDH) and glutamate dehydrogenase (GDH) in tissue homogenates of symbiotic and bleached *Isopora palifera* with or without nutrient supplementation.

Treatment	MDH	GDH	Free amino acids	
			Total	Essential
	(nmole NAD(P)^+^ min^−1^mg^−1^)		(pmole mg^−1^)	
Symbiotic control	77±18^a^ (14)	40±6^a^ (13)	385±43^a^ (9)	103±15^a^ (9)
Apo-symbiotic host	86±6^a^ (9)	20±4^b^ (9)	213±21^b^ (11)	58±8^b^ (11)
Apo-symbiotic host fed nutrient A	109±26^a^ (5)	24±4^ab^ (5)	372±29^a^ (5)	94±11^a^ (5)
Apo-symbiotic host fed nutrient B	42±9^a^ (11)	41±5^a^ (8)	281±34^b^ (11)	80±8^ab^ (11)
	F_3,35_ = 2.331 P>0.05	F_3,31_ = 3.292 P<0.05	F_3,32_ = 5.864 P<0.01	F_3,32_ = 3.264 P<0.05

Essential amino acids followed the definition applied to the sea anemone *Aiptasia pulchella* [19]. Enzyme activities were determined as the amount of NAD(P)H (in nmol) converted to NAD(P) by 1 mg of protein in 1 min. Nutrient compositions of A and B are described in “Materials and Methods” and Table 1. Numbers in parentheses represent the number of colony replicates, and means followed by the same letter do not significantly differ at *p* = 0.05 (Fisher's least significance difference test). Data are the mean±S.E.

**Table 3:** Contents of free amino acids and activities of malate dehydrogenase (MDH) and glutamate dehydrogenase (GDH) in tissue homogenates of symbiotic and bleached *Stylophora pistillata*.

Treatment	MDH	GDH	Free amino acids	
			Total	Essential
	(nmole NAD(P)^+^ min^−1^mg^−1^)		(pmole mg^−1^)	
Symbiotic control	46±11 (12)	21±1 (12)	250±20 (12)	75±12 (12)
Apo-symbiotic host	91±6 (18)	21±2 (20)	271±11 (21)	95±8 (21)
	F_1,28_ = 12.948 *p*<0.001	F_1,30_ = 0.026 *p*>0.05	F_1,31_ = 0.004 *p*>0.05	F_1,31_ = 2.231 *p*>0.05

Essential amino acids followed the definition applied to the sea anemone *Aiptasia pulchella* [19]. Enzyme activities were determined as the amount of NAD(P)H (in nmol) converted to NAD(P) by 1 mg of protein in 1 min. Numbers in parentheses represent the number of colony replicates, and data are the mean±S.E.

**Figure 5:**
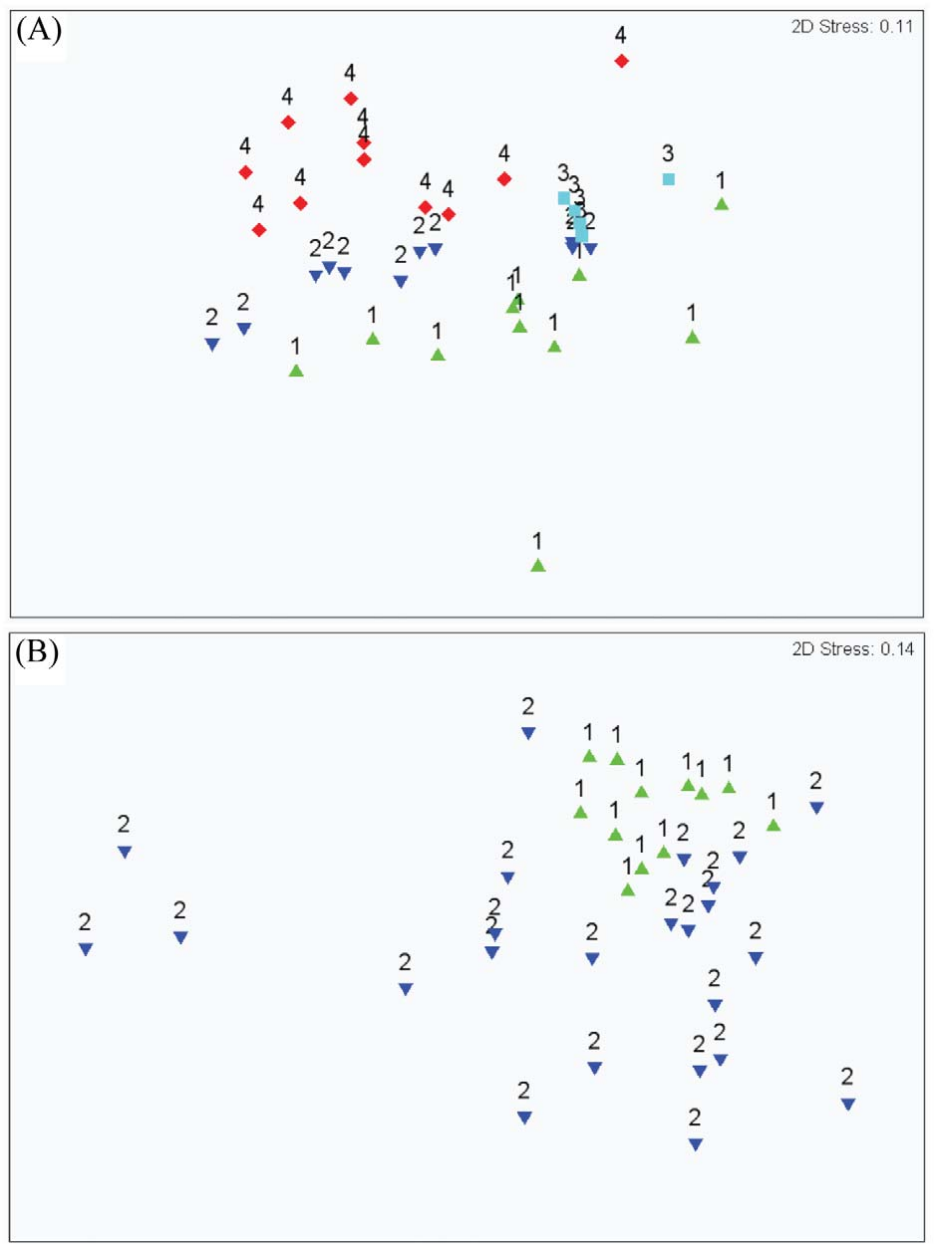
Multi-dimensional scaling (MDS) ordination of arc cosine-transformed free amino acid concentrations (mole%) in tissue extracts from symbiotic, bleached, and bleached coral with nutrient supplementation. (A) *Isopora palifera* (stress = 0.11); (B) *Stylophora pistillata*. (stress = 0.14). Data labels represent the treatment for coral (1, symbiotic control; 2, bleached coral; 3, bleached coral with nutrient A supplement; 4, bleached coral with nutrient B supplement).

A comparative analysis of FAAs indicated that dominant FAAs in the host homogenate of *Isopora* were glutamate, glycine, threonine, arginine, and lysine, which comprised >50 mol% of the total detected FAAs. When *Symbiodinium* was depleted from the host, threonine was the dominant FAA in the aposymbiotic host (25.6±4.7 mole%) and nutrient B-supplemented aposymbiotic host (20.4±3.0 mole%). The dominant FAA in the host homogenate of nutrient A-supplemented aposymbiotic *Isopora* was arginine (21.3±1.9 mole%) instead of the amino acids mentioned above. However, the dominant FAA in the host homogenate of *Stylophora* was aspartate for both the symbiotic (29.5±1.4 mole%) and aposymbiotic hosts (28.7±1.0 mole%). The variance analysis of the FAA composition further indicated that FAA profiles in the host homogenate significantly differed between symbiotic and aposymbiotic *Isopora* (with or without nutrient supplementation) (ANOSIM test, global *R* = 0.315, *p*<0.01, Fig. 5A), but no differences were found between symbiotic and aposymbiotic *Stylophora* (ANOSIM test, global *R* = 0.076, *p*>0.05, Fig. 5B). Pair-wise comparisons between different treatments in *Isopora* indicated a significant separation of FAA profiles in symbiotic coral from those in aposymbiotic coral (*R* = 0.205, *p*<0.01) and aposymbiotic coral supplemented with nutrient B (*R* = 0.466, *p*<0.001). However, the FAA profile in symbiotic *Isopora* could not be separated from that in the aposymbiotic counterpart supplemented with nutrient A (*R* = 0.122, *p*>0.05).

## Discussion

In this study, we applied menthol to develop a simple and gentle protocol to prepare aposymbiotic corals which retained comparable physiological and biochemical performances to their symbiotic counterparts by incubation in seawater only (for *Stylophora*) or with additional feeding of a nutrient cocktail containing glycerol, vitamins, and a host mimic FAA mixture (for *Isopora*). Bleaching coral by menthol, as indicated in Fig. 1B, occurred in a significant dose-dependent manner. However, because continuous incubation always caused high mortality, a repeated 8: 16-h menthol (treatment): ASW (resting) treatment cycle was essential for the success of the protocol (Fig. 2).

Menthol is a compound known to act on a variety of different membrane receptors, including the transient receptor potential (TRP)M8, TRPA1, and other ionotrophic receptors [39]. The binding of menthol to TRPM8 results in an increase in intracellular Ca^2+^ concentrations and causes a cold sensation in vertebrates [40]–[43]. Menthol was also found to cause antinociceptive and local anesthetic effects in neuronal and skeletal muscles via blocking voltage-operated sodium channels [44]. Menthol is also known to cause many adverse effects to plants, including photoinhibition [45]. In *Symbiodinium*-associated corals, the mechanism underpinning menthol-induced coral bleaching is not clear. However, based on two different *Symbiodinium*-releasing modes (ejecting the alga in a cloudy suspension by *Isopora* and releasing digested alga by *Stylophora*), the bleaching mechanism might be attributed to Ca^2+^-triggered exocytosis as described by Pang and Südhof [46] and/or photoinhibition in *Symbiodinium*. We have no information about Ca^2+^ movements in the coral host during menthol treatment, but a preliminary study indicated that menthol might inhibit *Symbiodinium* photosynthesis II activity in the millimolar range (4-h IC_50_ of 0.72∼1.96 mM) which was at a similar level that caused coral bleaching (unpublished data). Further studies are needed to clarify the mechanism of menthol-induced coral bleaching.

When depleting *Symbiodinium* from a cnidarian host, a cessation in the supply of photosynthate released from the algal symbiont would greatly upset the host physiology and metabolism. Although respiration rates of some corals (*Montastraea annularis*, *Agarwia lamarcki*, *Porites compressa*, and *Montipora capitata*) decreased when *Symbiodinium* algae were depleted [47], [48], those of freshly bleached *Isopora* and *Stylophora* did not significantly differ from the symbiotic counterparts (Fig. 4). No differences in respiration rates between symbiotic and aposymbiotic corals were found in the temperate coral *Astrangia danae*, which was interpreted as holozoic feeding in the aposymbiotic coral possibly compensating for the energy loss from the deprivation of photosynthate release by *Symbiodinium* [49]. Because no food sources are available in ASW, energy sources for the bleached *Stylophora* and *Isopora* to balance the loss from lack of photosynthate release by *Symbiodinium* might be derived from consuming previous reserves or digestion of impaired *Symbiodinium*.

Depletion of symbiotic algae would also result in significant changes in nitrogen metabolism of the host [25], [34], [50]. For example, GDH, a key enzyme for assimilating (or releasing) ammonium into (or from) amino acids, increases in alga-depleted corals and sea anemones [34]. FAAs, especially the so-called essential amino acids, in the host homogenates were also found to have decreased by nearly half after depletion of symbiotic algae [25], [50]. In this study, the responses of coral nitrogen metabolism to algal depletion differed between *Stylophora* and *Isopora*. Algal depletion caused significant decreases in *Isopora* GDH activity and FAA contents but not in *Stylophora*. However, supplementation of the aposymbiotic *Isopora* with nutrients containing glycerol, a host mimic FAA mixture, and vitamins reverted the nitrogen metabolic indices back to a level and composition comparable to the symbiotic counterpart (Table 2, Fig. 5A). This result is similar to previous findings in *Aiptasia* [25], [50]. Therefore, aposymbiotic coral generated by expelling *Symbiodinium* alive during bleaching would need to be fed a proper nutrient supplement before being subjected to physiological studies. With the nutrient A supplement, we successfully maintained *Isopora* for the test of reinfection with heterogenic *Symbiodinium* (unpublished data).

In summary, comparisons of physiological performances and gene expression profiles between different species of coral hosts per se will be available by preparing freshly bleached aposymbiotic coral with the menthol protocol combined with nutrient supplementation if necessary. This technique will also potentially benefit the search for a generalist coral to re-establish symbiosis with different heterogenic *Symbiodinium*, which will make the contributions of different *Symbiodinium* subclades to coral symbiosis more straightforward.
